# Creating a master training rotation schedule for emergency medicine residents and challenges in using artificial intelligence

**DOI:** 10.1186/s12245-024-00657-7

**Published:** 2024-07-04

**Authors:** Rawan Eskandarani, Ahmed Almuhainy, Abdulrahman Alzahrani

**Affiliations:** Emergency Medicine Administration, Second Health Cluster, King Fahad Medical City, Prince Abdulaziz Ibn Musaid Ibn Jalawi St., P.O. Box: 59046, Riyadh, 11525 Saudi Arabia

**Keywords:** Master rotation blocks, Emergency medicine board trainees, Rotation templates, Artificial intelligence role

## Abstract

**Background:**

The allocation of resident physicians to clinical rotations presents a complex challenge that requires balancing multiple objectives with the goals of providing optimal patient care, maintaining adequate departmental staffing, and maximizing residents’ training experience. While adhering to governing guidelines and training regulations, these physicians must comply with curricular milestones and educational goals for progression that must be achieved within specific time constraints. This manuscript reports on how to create annual master rotation schedules to meet the training requirements for 60 residents, while addressing the requirements detailed above.

**Methods:**

Trainees in the field of Emergency Medicine have to meet variable essential annual curricula requirements. Methods of preparing rotations in different Emergency Departments are presented which usually need early planning to ensure the best coordination and number allocation among the different internal and external collaborative departments. This off-institution and off-service external rotation is an educational necessity regulated by the Saudi Commission of Health Sciences to maximize residents’ exposure and meet the expected educational milestones unique to Emergency Medicine training.

**Results:**

We report how to create annual master rotation schedules to meet the training requirements for 60 Emergency Medicine residents, while maintaining steady adequate departmental staffing and accommodating the different external rotation capacities, a task that is usually handled by the chief residents and program director. Although some parts of this process can be performed by using scheduling software or with particular decision support management systems, many steps are still filtered and checked manually. External circumstances mandate changes in schedules that require last-minute changes which may overrule training restrictions and make them unfeasible.

**Conclusion:**

To create such an agile schedule, we maintain a standardized template with preset blocks and rotations. Residents can choose the most suitable track that meets their preference for year-long rotation blocks. Thus, we minimize the individual variability in the preset allocations, guarantee an even distribution, give equal chances to each resident to accommodate and approximate their individual preferences, and decrease the overall workload and time consumed annually.

## Introduction

The allocation of resident physicians to clinical rotations presents a complex challenge that requires balancing multiple objectives with the goals of providing optimal patient care, maintaining adequate departmental staffing, and maximizing the residents’ training experience. While adhering to governing guidelines and training regulations, resident physicians are required to comply with particular curricular milestones and educational goals for their progression that must be met within certain time constraints [1]. Additionally,, trainees in the field of Emergency Medicine have variable annual curricula that must be met. Their rotations in the different Emergency Departments both within the same institution and outside in collaborative institutions usually need to be planned before the beginning of the academic calendar to ensure the best coordination and number allocation among the different departments. This off-institution and off-service external rotation is an educational necessity regulated by the Saudi Commission of Health Sciences to maximize residents’ exposure and meet the expected educational milestones unique to Emergency Medicine training [2].

For the average training curriculum over a four-year program, a trainee is required to undergo series of main rotations in the Emergency Department, Pediatric Emergency, Intensive Critical Care, Cardiac Care Unit, Anesthesia, Orthopedics, Obstetrics and Gynecology, and Prehospital Medical Services. The annual rotation curriculum thus needs to be planned and set before the beginning of the academic year. The annual rotation schedule considers the number of trainees at each level. The maximum number of trainees that can be accommodated in each rotation ensures that every trainee completes their mandated rotations by the end of the year. In addition, while it is important to maintain a fair educational schedule, it is also important to consider that residents are not only trainees and many academic institutional structures rely heavily on residents as medical service providers. This needs to be considered to maximize patient care while complying with trainees’ working rules and hour restrictions to prevent fatigue and burnout [3, 4]. It is interesting to note that despite the commonly shared challenges regarding employee scheduling, few papers in the medical literature discuss these challenges or offer suggestions for best practices [5].

## Methods

Typically, the chief resident and program director manually allocate residents to different rotations. However, most errors affect the cost of rotation scheduling, and result in overstaffing or understaffing, thus compromising the residents’ optimal educational chances of exposure, and resulting in unequal distribution, increasing fatigue, rate of burnout, workload, and decreased overall satisfaction [3, 6]. The hard constraints that should lay the cornerstone for building any schedule start with a standardized total number of clinical shifts assigned to each level, limits on the total number of working hours, limits on the number of consecutive working shifts, restrictions on the total number of consecutive night shifts for each individual, restrictions on the number of weekends per month assigned to each resident, total vacation days, and distribution of these days between trainees over the year. Other factors to be considered and reasonably met are vacation timing, weekend and days’ off requests, and emergency leave [7, 8]. The schedule is typically set annually before the academic year begins and is based on the historical forecasting of trainee numbers, patient flow, departmental capacity, and seasonal changes. To add to the forecasting complexity, it is necessary to coordinate and collaborate with other specialty departments for off-service rotations and other emergency departments in different institutions.

### Where are we now?

Different approaches can be adopted depending on the size of the workplace, number of employees, working hours, areas that need to be covered, and patient load [5]. In nursing, mathematical programming using linear programming methods and heuristic artificial intelligence (AI) approaches have been suggested and used to solve and simplify this issue [9]. In the study, Sherali and colleagues reviewed scheduling issues and prior literature using goal programming to solve staff scheduling. They developed mixed-integer programming and heuristic solutions to address resident scheduling in hospitals. The three scenarios they focused their model programming on were: night work, departmental staffing and skill requirements, and residents’ preferences [10].

Several attempts have been made among different specialties based on the differing needs of the individual training programs in their corresponding hospitals and their available resources to solve resident scheduling issues [1, 2, 8, 11]. Although the proposed solutions are helpful and can establish future standards for setting resident schedules, many programs lack the resources to develop their own decision support systems or mathematical goal programming schedules. Hence we report our efforts that may assist other programs.

## Results

**Fig. 1:**
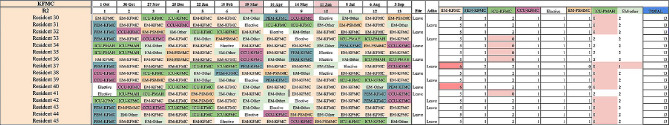
Master’s annual rotation schedule for Level 2 residents

Emergency Medicine residents in our training program totaled 63 trainees over a four-year program. They are required to complete a total of 26 blocks in Emergency Medicine, six blocks in Pediatric Emergency Medicine, two in the Intensive Care Unit, and one in each of the following: anesthesia, obstetrics and gynecology, orthopedics, cardiac intensive unit, Emergency Medical Services, Community Services, and Pediatric Intensive Care. In addition, six elective blocks are included, where the trainee can rotate according to their preferred specialty. Master Annual rotation schedules are created before the year begins, and allocations are distributed over the year to ensure fair distribution and fulfillment of requirements for each trainee based on their level (Fig. 1).

As we are working to create a more automated solution because of the scarcity of literature, we have included our spreadsheet workflow, which was developed and optimized over time to reach the best possible scenarios while continuing to move to the next phase of optimization.

### Rules for the spreadsheet

#### First layer

Whole-year rotation assignment was developed to ensure the completion for each resident of each of the levels’ requirements.

A master rotation sheet was created, where columns contain the block number with a total of 13 blocks (each block is 28 days regardless of the month), and rows are labeled with each resident’s name. All participants are grouped according to their corresponding year.

Fixed color coding for each rotation is used to identify missing rotations.

The number of required rotation columns are added to the end of the year columns to ensure each trainee has fulfilled the required curriculum by the end of the year.

This column has a set value for conditional formatting to change to red if it is not fulfilled by all the trainees.

The total number of rotations for each trainee was set to 13 and conditionally formatted to change to red if the specified value was not met.

Annual holiday blocks are labeled in red, and holiday dates should be considered when planning rotations and calculating staffing.

Holiday requests are considered during planning and added to ensure equal distribution while maintaining adequate staff coverage.

#### Second layer

Forecasting and planning a targeted desired number of residents in ‘your’ department while prioritizing the fulfilment of the required rotations.

**Fig. 2:**
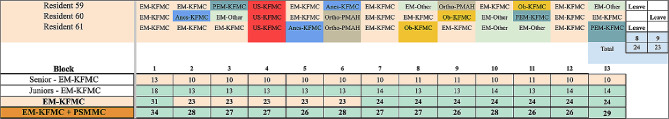
Forecasting and planning a targeted desired number of residents in your department while prioritizing the fulfilment of the required rotations

In the second planning layer, at the bottom of the master sheet, we used the (count if) function in the spreadsheet to determine the number of residents rotating in our department in a certain block. This was planned using historical forecasts of our department’s patient flow and seasonal changes (Fig. 2). The total number of trainees per block has has a set minimum number of residents. We allow for a wide range of flexibility to attain other hard constraints such as curricular requirements and vacation requests.

**Fig. 3:**
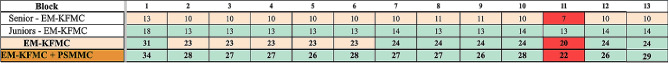
Forecasting and planning a targeted desired number of residents in your department while prioritizing fulfilling the required rotations. A deviation from the desired number is automatically highlighted in red

A soft constraint is added here, and we attempt to add a fair distribution of senior and junior levels. This is important for training purposes mainly because of the different levels of interaction, teamwork, supervision development, and leadership skills that need to be observed at the senior level. All residents are supervised by faculty regardless of their level of training. Conditional formatting is applied in all blocks for the minimal value to be highlighted in red if it was reached. In our case, we used the value of 21 (see Fig. 3).

### Third layer

**Fig. 4:**
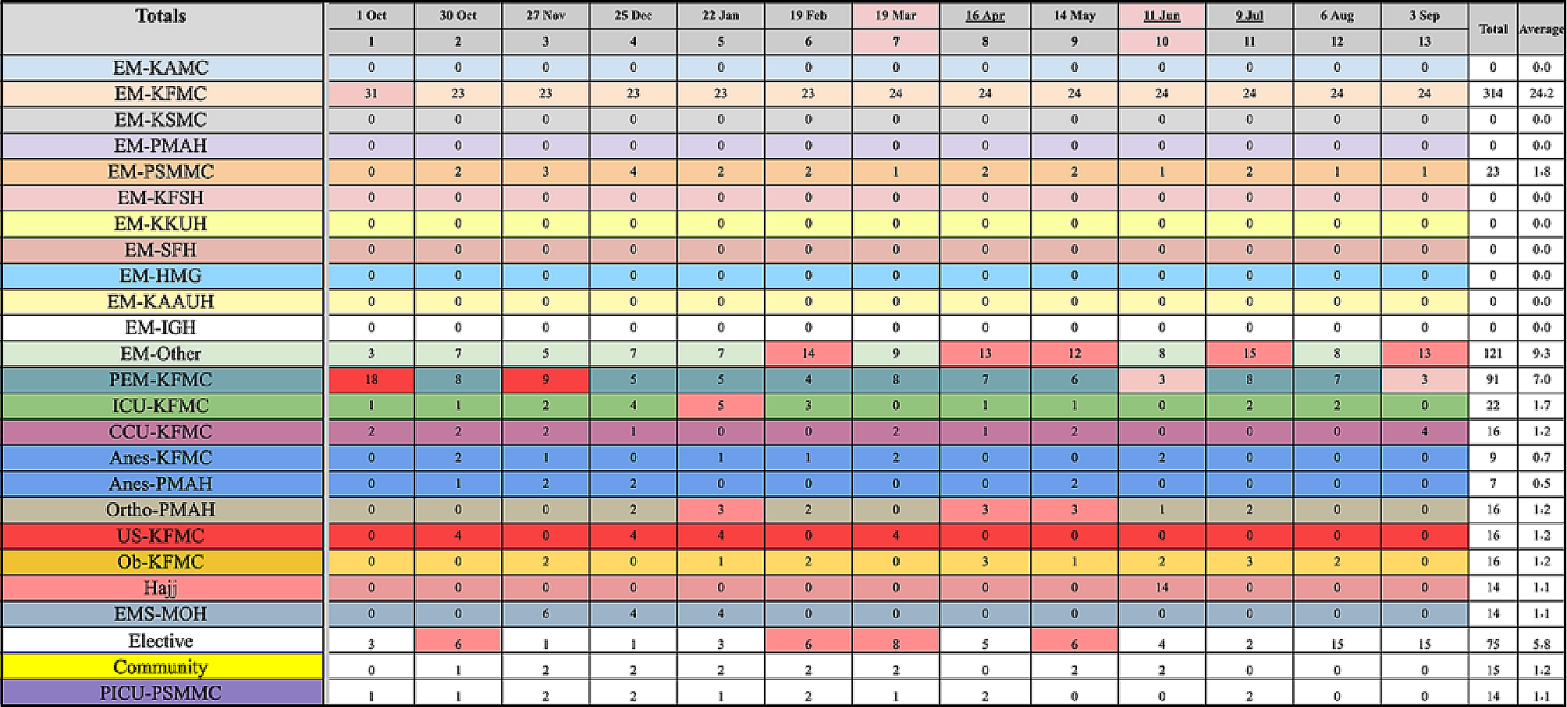
External rotation, planning, and even distribution

At the final end of the list, we use the same rules used above for color coding and assign minimal and higher value limits for each rotation in each block according to variable-affecting factors such as internal or external specialty availability, forecasted clinical exposure, and maximum capacity for visiting residents (Fig. 4). Many of these factors are not controlled and require a high level of flexibility and changes throughout the year. After setting the master rotation assignment, the infrastructure for the coming year is set.

**Fig. 5:**
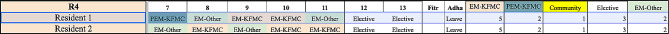
Added columns to the side of each resident show the total required rotations and target number of blocks to be completed by each resident each year

**Fig. 6:**
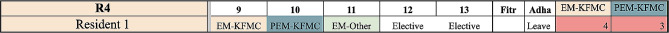
Rotations that exceeded or fell short of the set target for the required rotations per resident

**Fig. 7:**
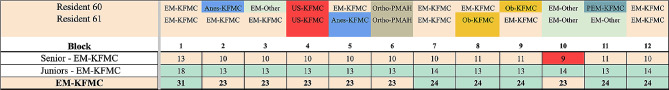
Example of falling short in the number of seniors in block number 10, automatically highlighted in red

**Creating a simple spreadsheet**:

- Create a spreadsheet with A columns for resident names.
- Each resident occupies a single row. Junior and senior levels have different color codes.
- Columns are created with block dates over the year (specify start and end dates).
- Color code dates of important holiday seasons to ensure proper staffing.
- Color code other important dates e.g. promotion exams.
- Color code each rotation to be unified across all schedules used.
- Columns are added at the end of each resident row filled with the rotations required for their level (Fig. 5).

- Use the formula (count if) in that row to ensure that each resident fulfills their level of required rotations for that year.
- Color data validation rules were used to create rules for the minimum and maximum target numbers for each rotation.
  - In the example above, an extra pediatric rotation (PEM-KFMC) was added instead of the (EM-KFMC).
  - The assigned target (PEM-KFMC) was 2 and (EM-KFMC) was 5.
  - It is automatically highlighted in red in both cells underneath the assigned rotations because there is an extra (PEM-KFMC) and a missing (EM-KFMC) (Fig. 6).

- Separate rows created at the end of the list of residents display the total number of residents assigned to each block.
  - This helps avoid clustering, as educational benefits and exposure decrease with the overcrowding of residents, especially if from similar levels.
  - Forecast your departmental staffing needs and patient exposure based on historical departmental patient flow and adjust if seasonal changes are expected.
  - Data validation and color highlighting are used to set the target number ranges so that any rotation changes in the above columns can be easily visually observed while trying to approximate the target ranges (Fig. 7).

- Creating a standard master’s rotation schedule while maintaining the flexibility to adapt to changes in external rotations, unforeseen trainees’ circumstances, unexpected discrepancies, and gaps, and being able to accommodate with minimal disruption to the rest of the rotation flow is necessary.
- To create such an agile schedule, we maintained a standardized template with preset blocks and rotations. Each resident can choose the most suitable track for year-long rotation blocks. Thus, we minimize individual variability in the preset allocations, guarantee an even distribution, give equal chances to each resident to accommodate and approximate their individual preferences, and decrease the overall workload and time to solve the manual rotation puzzle.

### Rules for the monthly residents rotation schedule

Following the same methods, our monthly schedules are planned, keeping in mind that resident fatigue and heavy workload are immediate consequences of a suboptimal schedule. Therefore, we try our best to accommodate requests, vacation requests, personal preferences, and unforeseen circumstances. Even soft constraints are considered where feasible.

### Challenges and suggested solutions

It is clearly not ideal to create the residents’ shift schedule manually. However, many programs still rely on either individual departmental team efforts or institutional-based expertise to solve this problem locally. Many mathematical wizards have addressed this employee-scheduling dilemma. Several solutions have been suggested and many institutions rely on commercial software for their employees and residents. Some better solutions can be costly in smaller institutions where hiring their own programming expertise is not feasible [10].

Most chief residents and program directors have certain bread-and-butter skills in handling such shift scheduling manually, while being conscious of common educational constraints and departmental allocation requirements along with training working hour restrictions [12]. Trying to write these constraints for any programming language makes one realize that there are more rules and constraints that they manually and casually check, which is not easy for any programming language to follow up and track.

In our program, spreadsheet templates were always used because the number of residents was limited. Over time, and with the growing number of residents, we started to face more difficulties and challenges in creating an annual resident rotation schedule that is closely linked to the resident’s monthly assignment, shift, and staffing allocation in the department. Realizing that human errors are inevitable, we had a larger team working and reviewing this process, and slowly learned different methods via trial and error with many tips and tricks that can be used in a spreadsheet to minimize human errors. As we prepare to use a decision-supporting linear programming system, we wanted to document and share our expertise since we realize that it is a constant dilemma faced by chief residents all over the world.

### Discussion of future possibilities

The Role of AI in the future:

It is interesting to navigate the rapid changes. We believe that AI’s will have a pivotal role in Shaping the Future, and It is anticipated to play a significant role in this domain to provide much simpler solutions.

With the recent rise of OpenAI’s Generative Pre-trained Transformer 3 (GPT-3), there is increasing interest in maximizing the benefits of using deep learning techniques. GPT-3 uses a neural network architecture to process sequential data to generate human-like responses with a pre-trained transformer. The model is pre-trained on large amounts of text data but can quickly adjust its parameters as input and generate a wider and more accurate response; the output is generated using a probabilistic approach pulled from its neural network.

*Utilizing Chat-GPT and maximizing its benefits.* It is crucial to provide the right input (prompt) and detailed data to allow it to fully explore, analyze, and uncover hidden patterns and trends in the data.

We understand the major limitations of this relatively new technology and do not believe that it will be a magical solution for creating employee shift schedules on demand. Experimenting with Chat-GPT has been very informative and we foresee that it could renovate the future.

Writing proper prompts improves the training of Chat-GPT and advances it to learn more about your data and context. Rules and constraints detailed in your data will help determine the most relevant outcomes for your input questions.

Based on your responses, two approaches can be taken:

1. Ask Chat-GPT to write a code snippet for use in the spreadsheet to create a shift schedule based on the data and inputs.
2. Ask Chat-GPT to create a schedule that you can copy and paste directly onto your spreadsheet.

A simple and straightforward schedule can be built if the number of employees is minimized. As the number of employees increases, more glitches start to appear. There were clear and unexplained inconsistencies that required us to repeat the experiment several times, indicating that it is not yet suitable for handling large datasets. Automatic General-Purpose Technology (AutoGPT) also promises to have a higher level of outcome and generated responses. It uses reinforcement learning and other machine learning techniques to search for the most efficient and effective architectures of the GPT family of language models.

One of the major limitations inherent to AI lies in its ability to comprehend pre-generated data sets designed for rotations and training Making it difficult to adapt to diverse scheduling scenarios. we found it to be much more helpful to specify the task or constrain on an already created schedule to clarify, filter, and remove duplicates, which ChatGPT and similar language models excel when you provide a specific prompt on an existing data as it lacks real-time information and excel in computational algorithms to validate an existing dataset and this can be more efficient the larger the dataset fed to the computational learning machine to create more reliable algorithms.

While this technology is not ready for full fledging. It is still very handy in its current versions and can help minimize significant time spent even by the average spreadsheet user, as you can use it as your own personal assistance tool that can perform specified complex tasks.

In our conducted series of experiments, we share our preliminary understanding of the potential advantages and challenges of using Chat-GPT in this context.The insights we share are by no means meant to be solid quantitative conclusions, but rather to emphasize the need for more controlled experiments. We aim to shed light on some hurdles and challnges we faced such as logistics and financial constraints to provide the necessary computational and expertise resources. Guidance, computational experts, and further rigorous research are highlights we believe should be emphasized.

## Conclusion

The complexity of creating resident scheduling that fulfills educational and patient care demands is a problem that is often tackled but still presents a challenge. The added challenges, working hour restrictions, and constraints make the suggested linear programming algorithms for resident schedules even more challenging than those for regular employees’ shift schedules. High levels of experience in mathematical computational programming and the rise of AI can pave the way in the near future to solve these complex problems and hopefully create generalizable algorithms and decision support systems that are user-friendly and customizable for application in any training program.

## Data Availability

The data supporting the findings of this study are available within the article.

## Abbreviations

AI: Artificial intelligence
AutoGPT: Automatic General-Purpose Technology
GPT-3: Generative Pre-trained Transformer 3
